# Cardioprotective Activities of Ethanolic Extract Root of *Ageratum conyzoides* on Alloxan-Induced Cardiotoxicity in Diabetic Rats

**DOI:** 10.1155/2020/3189672

**Published:** 2020-11-19

**Authors:** Abdulfatai Ojewale, Sanusi Mada, Samson Oyebadejo, Adam Afodun, Okikioluwa Aladeyelu, Bolaji Kolawole

**Affiliations:** ^1^Department of Anatomy, Faculty of Biomedical Sciences, Kampala International University, Western Campus, Bushenyi, Uganda; ^2^Department of Biochemistry, Faculty of Biomedical Sciences, Kampala International University, Western Campus, Bushenyi, Uganda; ^3^Department of Medical Laboratory Sciences, Faculty of Allied Health Sciences, Kampala International University, Western Campus, Bushenyi, Uganda; ^4^Department of Clinical Anatomy, Nelson Rolihlahla Mandela School of Medicine, KwaZulu-Natal University, Durban, South Africa

## Abstract

Diabetes mellitus has developed into one of the debilitating diseases disturbing the health of many people living with cardiovascular diseases in modern times. The root of *Ageratum conyzoides* was investigated for its effects on alloxan-induced diabetic Wistar rats' cardiac tissues. Thirty-two (32) Wistar rats weighing between 180 and 190 g were randomly divided into four groups. The animals in groups B-D were induced with a single dose of 150 mg/kg body weight of alloxan (ALX) intraperitoneally. They were confirmed hyperglycemic after 72 hours of induction and then sustained in hyperglycemic condition for 2 weeks. Animals in groups C and D received AC intervention, as stated above, for four weeks. The body weight of the experimental animals and blood collection for glucose estimation were taken weekly for six weeks using appropriate instruments. Biochemical assays for lipid profile, antioxidant enzymatic, and nonenzymatic markers were carried out. Histopathological changes in the cardiac tissues were also studied. Administration of 150 mg/kg of ALX to experimental rats induced diabetes and significantly reduced the body weights, significantly (*p* < 0.05) increased the glucose level, triglyceride (TG), total cholesterol (TC), and low-density lipoprotein (LDL) levels, and decreased the levels of high-density lipoprotein (HDL) and antioxidant enzymatic markers such as catalase (CAT), superoxide dismutase (SOD), and glutathione peroxidase (GPx) while the antioxidant nonenzymatic marker such as malondialdehyde (MDA) level was significantly increased. By contrast, rats given the ethanolic extract root of *A. conyzoides* had significantly (*p* < 0.05) increased the body weight gain, whereas the glucose levels significantly (*p* < 0.05) improved in treated diabetic rats. This extract also improved the cardiovascular system of the diabetic rats by significantly decreasing TG and LDL levels, significantly (*p* < 0.05) increasing the HDL level, significantly reducing the cardiac contents of CAT, SOD, and GPx, and significantly (*p* < 0.05) decreasing MDA. Ethanolic extract root of *A. conyzoides* exhibited antihyperglycemic and antihyperlipidemic activities and mitigates damage to the heart from the ALX-induced myocardial toxicity associated with type-1 diabetes.

## 1. Background

Diabetes mellitus (DM) is a persistent metabolic disorder associated with carbohydrate, lipid, and protein metabolisms that contribute to several kinds of complications, including diabetic cardiomyopathy [1]. Diabetic cardiomyopathy is one of the significant complications of diabetes mellitus. Diabetic cardiomyopathy is the most imperative basis of death in 65% of patients with diabetes [1].

Hyperglycemia and hyperlipidemia are the most critical risk factors for cardiovascular disorders [1]. The group of lipid abnormalities was coupled with diabetes with the occurrence of a high concentration of triglycerides (TGs), low concentration of high-density lipoprotein (HDL), and small dense low-density lipoprotein (LDL), and plasma cholesterol levels is generally expected [2]. Persistent hyperglycemia in the course of DM contributes to diabetic complications characterized by overproduction of reactive oxygen species (ROS) and buildup of lipid peroxidation by-products [3].

Experimental diabetes induced by alloxan (ALX) selectively disrupts the pancreas *β* cells, which are known to be one of the weakest structures to oxidative stress by generating excess reactive oxygen species and produces heart lesions that are similar to human diabetic cardiomyopathy [4]. Oxidative stress contributes to increase protein, lipid, and carbohydrate metabolisms, which links with increased free radical release accompanied by a decrease in antioxidants, leading to diabetes [5]. The rate of hyperglycemia and hyperlipidemia has been extensively documented and is implicated in the pathogenesis of various cardiovascular complications, including cardiomyopathy [1, 6].

Nutraceutical therapies for diabetes mellitus have established growing consideration during topical times, and various antioxidants, nutritional approaches, and medicinal plants have been anticipated for the management of metabolic disorders in diabetic patients [6]. *A. conyzoides* belongs to the Asteraceae family. It is an annual herbaceous plant that is widely circulated tropically and commonly used in Southern Nigeria. It is a scrub with a broad crown, fissured bark, and scented white flowers [7]. The plant is commonly found in West Africa and abundant, particularly in the Southern part of Nigeria. It is located in the savannah regions and swampy areas of Nigeria. It is generally called goat weed in English, Imi Esu in Yoruba, Igwulube in Igbo, Alkama Tuturuwa in Hausa, Otiti in Efik, and Nnyano in Ibibio. The leaves and roots of *A. conyzoides* (AC) are used in India and Brazil to treat fever and gastrointestinal diseases such as diarrhea, dysentery, rheumatism, ovarian inflammation, and intestinal colic with flatulence [8, 9].

In Cameroon and Congo, the leaves of AC are also used traditionally to treat fever, rheumatism, headache, and colic [10, 11]. In Central Africa, it is used traditionally to treat pneumonia; it is also used as an insecticide and nematicide [12]. The leaves are also used in dressing wounds and burns, and it has been shown to exhibit antibacterial activity [12, 13]. In reunion, the whole plant is used as an antidysenteric activity [14]. The whole plant has been used to treat colic, colds, rheumatism, spasms, and diarrhea, and it is also used as a tonic [15].

In Nigeria, the leaves and roots are useful in treating boils, leprosy, skin diseases, eye pains, and inflammation [16]. It has been shown to possess antidysenteric [17], analgesic [18], fertility, antispasmodic, and muscle relaxation properties [19, 20]. It has also been shown to possess anti-inflammatory and antipyretic properties [21]. The leaves and roots are also useful in treating hepatitis, breast myiasis sores, and arthritis [21, 22]. It has been reported to exhibit radioprotective, cardioprotective, and hepatoprotective properties [23–25]. Phytochemical screenings have shown that *A. conyzoides* extract root contains various phytochemical constituents, including flavonoids, saponins, tannins, terpenoids, alkaloids, and cardiac glycosides and does not contain anthracene derivatives [14, 17, 25].

Several antidiabetic plants have revealed an ameliorative effect on the cardiovascular system [1, 6]. The leaves of *A. conyzoides* were a potential source of antidiabetic agents [26]. Despite extensive natural use of *A. conyzoides* in traditional medicine for DM and other diverse ailments, its cardioprotective effects on the persistent metabolic disorder have not been scientifically validated. Therefore, the present study was designed to investigate the cardioprotective effects of ethanolic extract root of *A. conyzoides* on cardiac damage in the metabolic disorder of ALX-induced rats.

## 2. Materials and Methods

### 2.1. Sources of Chemicals, Reagents, and Kits

Alloxan (ALX) was purchased from a Sigma company representative in Nigeria, Glucometer kits were also purchased from the Fine test representative in Lagos, Nigeria (Fine-test®, Infopia Diagnostics). All other chemicals and reagents used in this study were of analytical grade.

### 2.2. Plant Material

The roots of *A. conyzoides* were collected from farmland in Ijebu-Ilugun, Ogun State, Nigeria, in the month of April 2016. The plant sample was identified and authenticated in the Forestry Research Institute of Nigeria (FRIN), Ibadan, Nigeria. The voucher specimen was deposited in the herbarium with a voucher number (FHI: 111925).

#### 2.2.1. Preparation and Extraction of the Plant Material

The roots of *A. conyzoides* were washed, cut into small pieces, sun-dried at room temperature for seven days, and crushed to a coarse powder with the electric marker. The powder (2500 g) was extracted with 96% absolute alcohol in 3 cycles using a soxhlet extractor. The crude extract was filtered with filter paper (Whatman No. 4). The filtrate was dried by a rotary vacuum evaporator at 30°C to obtain 387.2 g dry residue, a viscous brownish-coloured extract. It was stored in an airtight bottle kept in a refrigerator at 4°C till used. The extracted root was then reconstituted in distilled water at a suitable concentration for the experiment. Ojewale et al. [25] stated in their work that the LD_50_ of *A. conyzoides* was 5000 mg/kg body weight administered intraperitoneally; hence, the dosage used equivalent to 1/10^th^ dose (500 mg/kg body weight) was selected as the highest extract dose. The dose used was consistent with the previous investigation on the plant [25].

### 2.3. Experimental Animals

Thirty-two (32) healthy Wistar rats weighing between 180 and 190 g of either sex were obtained from the Laboratory Animal Center of College of Medicine, University of Lagos, Idi-Araba, Lagos, Nigeria. The rats were housed in clean, sharp cages and kept in a spacious room under room temperature at the Animal House of Faculty of Basic Medical Sciences, Obafemi Awolowo College of Health Sciences, Olabisi Onabanjo University, Ikenne, Ogun, Nigeria.

They were fed with standard animal pellets obtained from Pfizer Feeds Plc., Nigeria, and had access to water *ad libitum*. They were allowed to acclimatize for 14 days to the laboratory conditions before the experiment. The principles governing the use of laboratory animals as laid out by the Obafemi Awolowo College of Health Sciences, Olabisi Onabanjo University Research Ethical Committee were approved with the ethical number 06-08-2017-18, and the use and care of the animals and the experimental protocol were in strict conformity with the Institute of Laboratory Animal Research (ILAR) guidelines [27].

### 2.4. Induction of Experimental Diabetes

ALX was prepared in fresh normal saline. Diabetes was induced by intraperitoneal (ip) injection of alloxan monohydrate (150 mg/kg bwt) in a volume of 3 mL [28]. After 72 h, blood was withdrawn for blood glucose estimation monitored with a glucometer (Fine-test®, Infopia Diagnostics). The animals with ≥250 mg/dl blood glucose levels were considered diabetic and included in the experiment [29]. The diabetic animals were randomly distributed into three groups of eight animals each while the last group, the positive control, had eight normal rats (Table 1).

### 2.5. Weight Variation

At the outset, the animals were weighed and then weighed every seven days from the treatment's commencement until the 42nd day.

### 2.6. Determination of Fasting Blood Glucose

The diabetic rats were randomized into three (3) groups with eight (8) rats each; group B served as diabetic untreated control while groups C and D received graded doses of the extract by gavages and group A received normal saline once daily. The hyperglycemia was sustained and stabilized for two weeks, and the treatment was continued for four weeks. The blood samples were collected from the rat's lateral end of tail every week for six weeks and analyzed for glucose by oxidase method [30].

### 2.7. Animal Sacrifice

After six weeks of treatments (7 days after the experimental period), the animals were anesthetized with diethyl ether. The blood was collected through the cardiac puncture into sample bottles devoid of the anticoagulant. The samples were centrifuged at 4000 rpm for 5 minutes to obtain the sera. The abdominal cavity of each rat was opened up through a midline abdominal incision to expose the heart. The cardiac tissue was excised and weighed; the cardiac tissue was weighed electronically and balanced. Blood was collected with a heparinized tube and was centrifuged within 10 minutes of the collection at 4000 rpm for 5 minutes to obtain blood plasma was analyzed for lipid profile. Each animal's cardiac tissue was fixed in Bouin's fluid for 48 hours before the histological procedure commenced.

### 2.8. Assay of Lipid Profile

Total cholesterol (TC), total triglyceride (TG), and high-density lipoprotein-cholesterol (HDL-Chol) levels were determined by using previously modified enzymatic procedures [31]. Low-density lipoprotein-cholesterol (LDL-Chol) levels were calculated using the Friedewald equation [32].

### 2.9. Evaluation of Enzymatic Antioxidants

#### 2.9.1. Determination of Catalase (CAT) Activity

Catalase activity was evaluated according to the method previously described by Aebi [33]. Activity of catalase was expressed as unit mg^−1^ protein.

#### 2.9.2. Determination of Superoxide Dismutase (SOD) Activity

Superoxide dismutase activity was evaluated according to the method previously described by Winterbourn et al. [34]. It was expressed as u mg^−1^ protein.

#### 2.9.3. Determination of Glutathione Peroxidase (GPx) Activity

Glutathione peroxidase activity was determined by the method previously described by Rotruck et al. [35]. The absorbance of the product was read at 430 nm, and it was expressed as nmol^−1^ protein.

### 2.10. Evaluation of Nonenzymatic Antioxidant

#### 2.10.1. Determination of Lipid Peroxidation (Malondialdehyde)

Lipid peroxidation was estimated colorimetrically by the thiobarbituric acid reactive substance (TBARS) method previously described by Buege and Aust [36]. Concentration was measured using the molar absorptive of malondialdehyde, which is 1.56 × 105 M^−1^ cm^−1^, and it was expressed as nmol mg^−1^ protein.

### 2.11. Histopathological Analysis

On the last day of the experiment, the cardiac tissues were removed from each group and were fixed in Bouin's fluid for 48 hours. Histopathological analysis was done as previously described by [28].

### 2.12. Statistical Analysis

Results were analyzed and presented as the mean ± standarderrorofthemean (SEM) using GraphPad Prism 5.01. Analysis of variance (ANOVA) and Turkey's post hoc test were employed to test the significance of difference across the groups, and *p* < 0.05 was considered statistically significant.

## 3. Results

### 3.1. Effect of Ethanolic Extract Root of *A. conyzoides* on Body Weight (g) in ALX-Induced Diabetic Rats

The weight of animals in groups A, C, and D (Table 2) during the administration progressively increased weekly throughout the administration period. These are contrary to the animals in group B with a progressive weight loss weekly. Also, animals in group B showed a progressive weight loss (from 198.6 ± 10.9 g to 162.8 ± 10.1 g); this implies that they lost weight when the animals' initial value in 1st week and final weight of the animals in 6th week (-35.8) were compared. However, there was an increased in the weight gain of animals in the groups A, C, and D when their (1st week) initial weight (188.4 ± 8.6 g, 195.2 ± 9.8 g, and 192.2 ± 8.8 g) was compared with the (6th week) final weight (212.6 ± 10.8 g, 212.4 ± 7.5 g, and 221.0 ± 8.5 g).

### 3.2. Effect of Ethanolic Extract Root of *A. conyzoides* on Blood Glucose Level (mg/dl) in ALX-Induced Diabetic Rats

In this present study, it was revealed that a study of the effect of the extracts on fasting blood sugar levels of diabetic rats is shown in Table 3. As indicated, the treatment of ALX-induced diabetic rats with graded doses of the root extract caused a significant (*p* < 0.05) decrease in the animals' blood sugar concentration. At postinduction, the measurement of the blood glucose levels was on the high side. It was evident after the second week through to the last day of oral administration of *A. conyzoides* at both 250 or 500 mg/kg body weight, and the extract exhibited a significant (*p* < 0.05) reduction in blood glucose level.

### 3.3. Effects of the Ethanolic Extract Root of *A. conyzoides* on Lipid Profile Level ALX-Induced Diabetic Rats

In this study, it was observed that total cholesterol was significantly higher when rats became diabetic. In the diabetic treated groups with the ethanolic extract root of *A. conyzoides* (Table 4), a significant (*p* < 0.05) reduction in plasma cholesterol level was observed in experimental compared to the diabetic untreated group. The higher dose of the extract of 500 mg/kg (93.6 ± 1.2) exerted a more significant (*p* < 0.05) reduction compared to the 250 mg/kg (98.4 ± 1.6). There was a gradual decrease in triglyceride levels in the *A. conyzoides*-treated groups against the diabetic untreated group. A significant (*p* < 0.05) increase was observed in diabetic treated groups' triglyceride levels compared to normal control and diabetic groups.

There was a gradual decrease in LDL levels in the *A. conyzoides*-treated groups as against the untreated diabetic group. A significant (*p* < 0.05) increase was observed in LDL levels in diabetic treated groups compared to the normal control group and diabetic group. There was an increase in HDL levels in all the treated groups as against the untreated. A significant (*p* < 0.05) decrease was observed in HDL levels in the diabetic untreated group compared to the normal control.

### 3.4. Effects of Ethanolic Extract Root of *A. conyzoides* on Pancreatic Antioxidant Enzymatic and Nonenzymatic Markers in ALX-Induced Diabetic Rats

**Figure 1:**
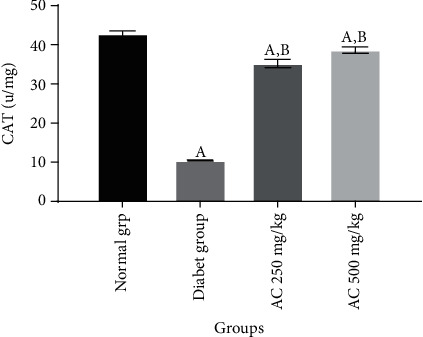
Effect of ethanolic extract root of *A. conyzoides* on cardiac enzymatic antioxidant level (CAT u/mg) across the groups. Values represent mean ± SEM; *n* = 8. Group A: consisting of control rats; group B: consisting of diabetic rats; group C: consisting of treated diabetic rats received 250 mg/kg of *A. conyzoides*; group D: consisting of treated diabetic rats received 500 mg/kg of *A. conyzoides.*^a^Statistically significant when compared to the normal (control) group at *p* < 0.05. ^b^Statistically significant when compared to the diabetic untreated group at *p* < 0.05.

**Figure 2:**
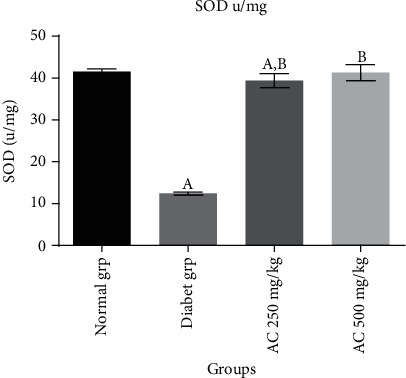
Effect of ethanolic extract root of *A. conyzoides* on cardiac enzymatic antioxidant level (SOD u/mg) across the groups. Values represent mean ± SEM; *n* = 8. Group A: consisting of control rats; group B: consisting of diabetic rats; group C: consisting of treated diabetic rats received 250 mg/kg of *A. conyzoides*; group D: consisting of treated diabetic rats received 500 mg/kg of *A. conyzoides.*^a^Statistically significant when compared to the normal (control) group at *p* < 0.05. ^b^Statistically significant when compared to the diabetic untreated group at *p* < 0.05.

**Figure 3:**
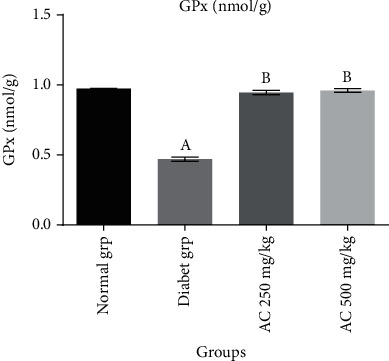
Effect of ethanolic extract root of *A. conyzoides* on cardiac enzymatic antioxidant level (GPx nmol/g) across the groups. Values represent mean ± SEM; *n* = 8. Group A: consisting of control rats; group B: consisting of diabetic rats; group C: consisting of treated diabetic rats received 250 mg/kg of *A. conyzoides*; group D: consisting of treated diabetic rats received 500 mg/kg of *A. conyzoides.*^a^Statistically significant when compared to the normal (control) group at *p* < 0.05. ^b^Statistically significant when compared to the diabetic untreated group at *p* < 0.05.

**Figure 4:**
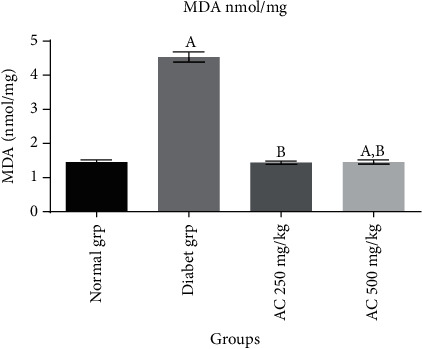
Effect of ethanolic extract root of *A. conyzoides* on cardiac nonenzymatic antioxidant level (MDA nmol/mg) across the groups. Values represent mean ± SEM; *n* = 8. Group A: consisting of control rats; group B: consisting of diabetic rats; group C: consisting of treated diabetic rats received 250 mg/kg of *A. conyzoides*; group D: consisting of treated diabetic rats received 500 mg/kg of *A. conyzoides*. ^a^Statistically significant when compared to the normal (control) group at *p* < 0.05. ^b^Statistically significant when compared to the diabetic untreated group at *p* < 0.05.

In this study, it was established that diabetic untreated group (Figures 1–3) showed a statistically significant decrease (*p* < 0.05) in CAT (10.8 ± 0.6 u/mg), SOD (12.8 ± 0.2 u/mg), and GPx (0.48 ± 0.6 nmol/mg) activities compared to normal rats without treatment. Diabetic rats treated with *A. conyzoides* significantly increased (*p* < 0.05) in cardiac CAT (36.4 ± 1.1 to 38.2 ± 1.3 u/mg), SOD (38.2 ± 1.2 to 40.8 ± 1.4 u/mg), and GPx (0.96 ± 0.5 to 0.97 ± 0.4 nmol/mg) activities (Figures 1–3) compared to the diabetic untreated animals, along the same line. However, a significant decrease (*p* < 0.05) in MDA content (4.4 ± 0.6 nmol/mg) (Figure 4) was observed in the diabetic untreated group when compared to the normal animals. Diabetic treated group with the *A. conyzoides*, however, significantly reduced the MDA (1.48 ± 0.2 to 1.47 ± 0.4 nmol/mg) (Figure 4) compared to the diabetic group.

### 3.5. Effects of Ethanolic Extract Root of *A. conyzoides* on Pancreatic Antioxidant Nonenzymatic Markers in ALX-Induced Diabetic Rats

It was observed that the diabetic untreated group showed a significant decrease (*p* < 0.05) in MDA content (4.4 ± 0.6 nmol/mg) compared to the normal animals (1.54 ± 0.2 nmol/mg). Diabetic treated group with the *A. conyzoides*, however, significantly reduced the MDA (1.48 ± 0.2 to 1.47 ± 0.4 nmol/mg) (Figure 4) compared to the diabetic group.

### 3.6. Histopathological Findings

The photomicrograph of the normal heart (A) group showed normal myocardial and normal widening of interstitium of the heart tissue. The photomicrograph of the diabetic untreated (B) group of heart tissue revealed myocardial distortion, characterized with cellular degeneration and reduction of interstitium of the heart. The photomicrograph of the diabetic treated with 250 mg/kg of *A. conyzoides* (C) showed that there was a slight recovery of myocardial tissue with the interstitium of the heart tissue, while the photomicrograph of the diabetic treated with 500 mg/kg of *A. conyzoides* (D) showed complete restoration of myocardial tissue following the reversal of the degenerative changes and widening the space of interstitium of the heart tissue.

## 4. Discussion

In this present study, it was revealed that blood glucose levels significantly increased. Still, body weight gain decreased after injection of ALX to the rats, contrast blood glucose, triglyceride, total cholesterol, LDL, SOD, CAT, and GPx and had a significant decrease by distinguishing body weight; HDL and MDA had a considerable increase in diabetic rats without treatment.

Administration of 150 mg kg^−1^ of ALX to rats induced experimental diabetes and significantly reduced the body weight, which confirms the induction of diabetes compared with the diabetic treated and normal control rats. ALX-induced experimental diabetes causes a significant weight loss in body weight while treatment with *A. conyzoides* extracts significantly increases body weight. Bodyweight loss is one of the physical manifestations of diabetes that occurs due to chronic hyperglycemia, resulting in muscle wasting and loss of tissue protein and leads to increased protein glycation [37].

Oral administration of ethanolic extract root of *A. conyzoides* to the diabetic rats at the dose of 250 and 500 mg/kg significantly (*p* < 0.05) decreases blood glucose level in a dose-dependent manner; diabetic rats without treatment showed a numerical increase in blood glucose level compared to the normal rats. Our finding is in accordance with the reports of [25, 26].

Ethanolic extract root of *A. conyzoides* has been shown to possess the following phytochemical constituents: tannins, reducing sugars, saponins, alkaloids, terpenoids, flavonoids, steroid glycosides, and carbohydrate. Flavonoids, saponins, and terpenoids have been implicated to have known to be bioactive substances against diabetes [25, 39].

The significant reduction of glucose concentration could partly be that the phytochemical constituents of *A. conyzoides* promoted glucose entry into cells to reduce the release of glucose in the blood. This study showed that ALX-induced diabetes led to various lipid abnormalities, and it has been reported in previous studies that the activities of ALX caused lipid profile derangement [30, 38, 39]. The marked increase in the high concentration of serum lipids was mainly due to the activation of the hormone-sensitive lipase during insulin insufficiency, which may be due to the rise in the release of fatty acids from peripheral tissue to the blood vessels. Diabetes is a debilitating disease associated with hyperglycemia characterized by dyslipidemia, which is a risk factor for coronary heart diseases [40].

When the experimental rats became hyperglycemic in lipid studies, there was an elevated level of total cholesterol, which was implicated along with the marked increase of triglyceride, which activates the enzyme lipoprotein lipase, an atherogenic pattern of risk factors that include a lower level of HDL-cholesterol. Higher LDL concentrations are predictive of coronary events independent of other coronary disease risk factors [41, 42, 43]. Several antidiabetic plants have been implicated in mitigating blood plasma lipid levels, usually curtailing the risk of exposure to cardiovascular disease [43–45]. This study established the efficacy of *A. conyzoides* root extract ameliorating hyperlipidemia. The subdued extract activation of the hormone-sensitive lipase during insulin insufficiency may be due to the rise in the release of fatty acids from peripheral tissue to the blood vessels. Our finding is in arrangement with the reports of Ahmed et al. [45], Mbaka et al. [46], and Udenze et al. [47].

It has been reported that the cytotoxic action of ALX is mediated by the formation of free radicals such as superoxide and catalase radicals, which selectively damages the *β* cells of the pancreas, which are known to be one of the weakest structures to oxidative stress by generating excess reactive oxygen species and produces heart lesions that are similar to human diabetic cardiomyopathy [4, 6].

**Figure 5:**
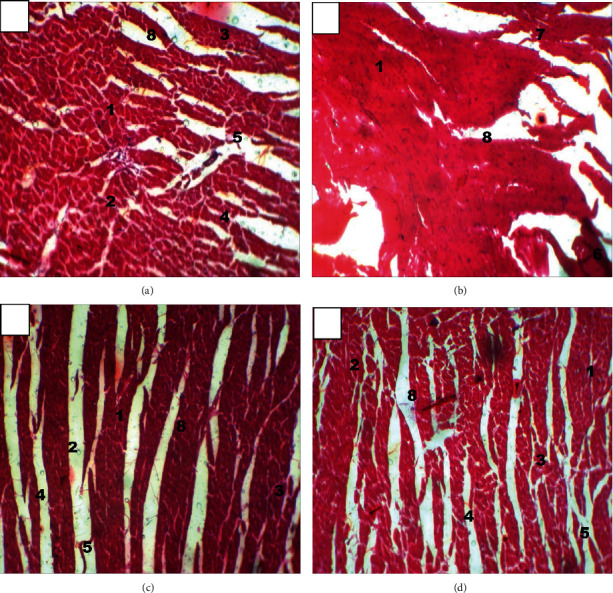
(a–d) The photomicrograph of the heart tissue in (a) normal control, (b) diabetic untreated, (c) diabetic treated with 250 mg/kg of *A. conyzoides*, and (d) diabetic treated with 500 mg/kg of *A. conyzoides* rats. Magnification ×100, stained with H & E. 1: cardiac muscle cells; 2: cardiac muscle nuclei; 3: intercalated discs; 4: striations; 5: myofibrils; 6: cellular degeneration; 7: myocardial distortion; 8: interstitium.

Excessive ROS production that exceeds critical levels can overcome all the heart's antioxidants' protection strategy, causing oxidative stress that damages the biological tissues in the hearts. This, in turn, may cause the degeneration of the myocardium, which distorts myocardial and reduces the interstitium space of the heart (Figure 5(b)) [4]. However, the extract suppresses ALX's cytotoxic action by causing the regeneration of the myocardium, which restores myocardial tissues and increases the heart's interstitium space, which was observed in the histoarchitectural analysis (Figure 5(c) and Figure 5(d)). Appraisal of SOD, CAT, GPx, lipid peroxidation, and other antioxidant enzyme activities in biologicaltissue has always been used as markers for oxidative stress [48, 49].

In this study, diabetic rats treated with the *A. conyzoides* markedly alleviate the oxidative damage by ALX induced in rats. It was observed that the administration of *A. conyzoides* upturned the enhanced lipid peroxidation and the consequent decline in the level of enzymatic antioxidant (GPx, SOD, and CAT) as well as the activities of nonenzymatic antioxidant (MDA) in the cardiac tissue of diabetic rats. Our finding is in accordance with the reports of [6, 28, 49, 50].

This study suggests that *A. conyzoides* does not have cardiotoxic effects. Still, relatively, it could potentially be further investigated for use in the management of the development of diabetes and improved the structure and functions of the heart.

## 5. Conclusion

Oral administration of ethanolic extract root of *A. conyzoides* exhibited antihyperglycemic and antihyperlipidemic activities and mitigates damage to the heart from the cardiac toxicity with ALX-induced diabetes. It could be used as an antidiabetic agent with a potent cardioprotective effect, and this effect may be attributed to a variety of phytochemical constituents present in *A. conyzoides.*

## Figures and Tables

**Table 1:** Grouping and treatments of the experimental animal.

Groups	Treatment
A	Normal control group administered with normal saline for 6 weeks.
B	Diabetic untreated group was induced with 150 mg/kg bwt of alloxan and was given normal saline for 6 weeks.
C	Diabetic untreated rats for 2 weeks; diabetic rats were then administrated with the 250 mg/kg bodyweight of *A. conyzoides* for 4 weeks.
D	Diabetic untreated rats for 2 weeks; diabetic rats were then administrated with the 500 mg/kg bodyweight of *A. conyzoides* for 4 weeks.

**Table 2:** Effect of oral administration of ethanolic extract root of *A. conyzoides* for six weeks on body weight (g) in ALX-induced diabetic rats.

Groups	Initial body weight (g)	Final body weight (g)	Difference in body weight (g)
A	188.4 ± 8.6	212.6 ± 10.8	24.2
B	198.6 ± 10.9	162.8 ± 10.1^∗^	-35.8
C	195.2 ± 9.8	212.4 ± 7.5^∗∗^	17.2
D	192.2 ± 8.8	221.0 ± 8.5^∗∗^	29.0

Values are the meanvalues ± standarddeviation of 8 rats; group A: consisting of control rats; group B: consisting of diabetic rats; group C: consisting of treated diabetic rats received 250 mg/kg of *A. conyzoides*; group D: consisting of treated diabetic rats received 500 mg/kg of *A. conyzoides*. ^∗^Statistically significant when compared to the control group (A) at *p* < 0.05. ^∗∗^Statistically significant when compared to the diabetic untreated group (B) at *p* < 0.05.

**Table 3:** Effect of ethanolic extract root of *A. conyzoides* on blood glucose level (mg/dl) in ALX-induced diabetic rats.

Time/groups	A	B	C	D
0 hour	96.4 ± 7.6	94.8 ± 8.4	92.8 ± 6.6	88.3 ± 4.2
72 hours	92.2 ± 6.9	496.6 ± 18.0	489.8 ± 15.4	492.2 ± 14.6
2 weeks	83.7 ± 8.6	518.6 ± 16.4	504.8 ± 12.4	512.6 ± 11.4
4 weeks	96.8 ± 7.4	497.2 ± 14.2	246.1 ± 11.8	227.8 ± 12.8
6 weeks	92.4 ± 8.4	488.6 ± 17.8	149.1 ± 14.5^a^	138.4 ± 13.4^a^

Values represent mean ± SEM; *n* = 8. Group A: consisting of control rats; group B: consisting of diabetic rats; group C: consisting of treated diabetic rats received 250 mg/kg of *A. conyzoides*; group D: consisting of treated diabetic rats received 500 mg/kg of *A. conyzoides.*^a^Statistically significant when compared to the control group (A) at *p* < 0.05.

**Table 4:** Effects of the ethanolic extract root of *A. conyzoides* on lipid profile level.

Group	Dose	Total cholesterol (TC)	Triglyceride (TG)	High-density lipoprotein (HDL)	Low-density lipoprotein (LDL)
A		92.4 ± 2.1	92.6 ± 1.1	28.4 ± 1.1	49.8 ± 0.4
B		321.6 ± 8.4^a^	374.7 ± 5.7^a^	14.3 ± 0.6^a^	187.4 ± 5.2^a^
C	250 mg/kg	98.4 ± 1.6^a,b^	98.9 ± 2.4^a,b^	18.6 ± 0.6^a,b^	61.2 ± 2.2^a,b^
D	500 mg/kg	93.6 ± 1.2^a,b^	95.8 ± 1.4^a,b^	26.5 ± 0.2^a,b^	53.6 ± 1.2^a,b^

Values represent mean ± SEM; *n* = 8. Group A: consisting of control rats; group B: consisting of diabetic rats; group C: consisting of treated diabetic rats received 250 mg/kg of *A. conyzoides*; group D: consisting of treated diabetic rats received 500 mg/kg of *A. conyzoides.*^a^Statistically significant when compared to the control group (A) at *p* < 0.05. ^b^Statistically significant when compared to the diabetic group (B) at *p* < 0.05.

## Data Availability

The datasets generated during the analysis used to support our findings of this study are available from the first and corresponding authors on reasonable requests.
